# An Outbreak of Hepatitis A among Young Adult Men in Cyprus

**DOI:** 10.3390/pathogens9110979

**Published:** 2020-11-23

**Authors:** Panagiotis Dimitriou, Georgios K. Nikolopoulos, Maria Koliou, Elisavet Constantinou, Chara Azina, Maria Panayiotou, Eirini Christaki

**Affiliations:** 1 Department of Medicine, Nicosia General Hospital, 2029 Nicosia, Cyprus; panagikd@auth.gr (P.D.); OKYPY77@hio.org.cy (C.A.); christaki.eirini@ucy.ac.cy (E.C.); 2 Medical School, University of Cyprus, 2029 Nicosia, Cyprus; dr.mariapanayiotou@gmail.com; 3 Unit for Surveillance and Control of Communicable Diseases, Medical and Public Health Services, Ministry of Health, 1448 Nicosia, Cyprus; mkoliou@spidernet.com.cy (M.K.); econstantinou@moh.gov.cy (E.C.)

**Keywords:** hepatitis A, outbreak, sexually transmitted diseases, men having sex with men

## Abstract

Background: Outbreaks of acute hepatitis A (AHA) have recently been reported in Europe among men who have sex with men (MSM). The aim of this work was to evaluate, for the first time, trends in the reported cases of AHA in Cyprus over the last seven years. Methods: We retrospectively studied all people reported with AHA in Cyprus between January 2013 and December 2019. Demographic data, type of transmission, vaccination status for HAV, laboratory and clinical data were analyzed. Results: The asnalysis involved 33 AHA cases (age 32.7 ± 17.4 years, 78.8% males). An increase in AHA reports was observed between July 2017 and June 2018 when more than a third (*n* = 13) of the cases of the period 2013–2019 were reported. The reporting rate of AHA doubled from 0.52 cases per 100,000 population (before July 2017) to 1.12 cases per 100,000 population (July 2017–June 2018). The male/female (M/F) ratio increased from one in 2013 to eight in 2018. Conclusion: An increase in AHA reports occurred in Cyprus between July 2017 and June 2018. Many cases with AHA in that period were MSM. Enhanced surveillance and timely public health interventions, like vaccination and awareness promotion, are important for preventing future outbreaks.

## 1. Introduction

Hepatitis A is an acute infection caused by the hepatitis A virus (HAV), which is primarily transmitted by the fecal–oral route, via person to person contact or by consumption of contaminated water or food [1]. Common types of transmission include consumption of uncooked or inadequately cooked food and raw shellfish, sexual contact (oral–anal contact), close contact with an infected person, injection of illicit drugs among infected persons, and, rarely, blood transfusion. Acute hepatitis A (AHA) is usually symptomatic in adults, causes jaundice and elevated liver enzymes, and most of the time is self-limited. However, fulminant hepatic failure can rarely occur, in less than 1% of patients, with a bad prognosis, often requiring hepatic transplantation. Patients with underlying hepatic disease, like those with chronic hepatitis B or C, have a greater risk for fulminant hepatitis [2].

The incidence of AHA has declined substantially over the last decades due to improvements in hygiene, regulations regarding the handling of food, and implementation of vaccination against HAV. Data from Europe show that the annual incidence has declined from 10 (1997) to 2.5 cases per 100,000 persons (2011) [3]. However, outbreaks of AHA continue to appear nowadays, including outbreaks in the community, due to food contamination, outbreaks among homeless people, and outbreaks in healthcare settings [4,5,6]. Recently, there have been several reports of outbreaks of AHA among men who have sex with men (MSM) in many European countries [7,8,9]. Two groups from France described such outbreaks in MSM living with HIV and MSM using pre-exposure prophylaxis (PrEP) [10,11]. These findings have prompted the European Center for Disease Prevention and Control (ECDC) and the US Centers for Disease Prevention and Control (CDC) to provide guidance on recent AHA outbreaks [12,13]. Moreover, a CDC Health Update underlined the importance of such outbreaks to public health [14].

Clinicians of our team observed a notable increase in hospitalized AHA cases in Cyprus during the second semester of 2017. Five young men with AHA needed hospital care in the summer of 2017 compared to only one to two cases of AHA who were hospitalized in our tertiary hospital before. In view of the recent outbreaks in Europe, we aimed to further evaluate this clinical observation.

## 2. Materials and Methods

We retrospectively studied all reported cases of AHA in Cyprus between January 2013 and December 2019. Data without personal identifiers were retrieved from surveillance records of the Unit for Surveillance and Control of Communicable diseases of the Cyprus Ministry of Health. The protocol of this work was approved by the Cyprus Bioethics Committee and the Committee for Research in Clinical Settings of the Cyprus Ministry of Health.

The case definition of confirmed AHA included a compatible clinical presentation (jaundice and abnormal liver function tests, abdominal pain, nausea, fatigue, fever) and laboratory-confirmed anti-HAV immunoglobulin M (IgM) serology.

Demographic data (sex, age, ethnicity, place of residence, recent travel), type of transmission (food-borne, contact with other cases, sexual, drug injection), vaccination status for HAV, laboratory data (serum glutamic acetoacetic transaminase (SGOT), serum glutamic pyruvic transaminase (SGPT), international normalized ratio (INR), bilirubin, creatinine, alkaline phosphatase (AP), gamma glutamic transferase (γ-GT), antibodies for human immunodeficiency virus (HIV), hepatitis B virus (HBV) and hepatitis C virus (HCV)), and clinical data (symptoms/sings, course of illness, duration of hospitalization, and prognosis) were analyzed.

## 3. Results

**Figure 1:**
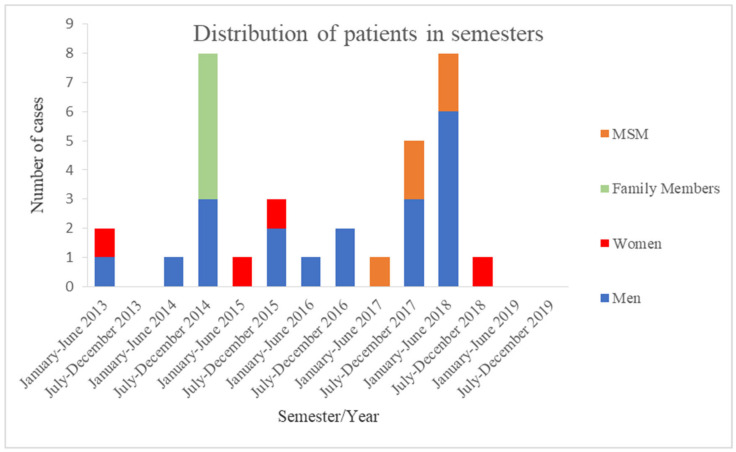
Distribution of patients with acute hepatitis A in semesters (2013–2019), MSM: men who have sex with men.

Between January 2013 and December 2019, 33 AHA cases were reported to the Unit for Surveillance and Control of Communicable diseases of the Cyprus Ministry of Health. Of interest, in one year, from July 2017 to June 2018 (outbreak period), 13 patients were diagnosed with AHA in Cyprus (5 during the second semester of 2017, 8 until 6/2018). The distribution of the 33 cases, both from the public and private health sectors, in the six-year period is presented in Figure 1. From June 2013 to June 2017, the reported numbers of patients with AHA per semester were low (from one to three reports/semester) except the second semester of 2014 when a family with five members (a mother and four children) and recent travel to Syria were diagnosed with AHA. The family returned to Cyprus around 40 days before symptom onset, a period within the incubation period of AHA (range 15 to 50 days). The reporting rate doubled from 0.52 cases per 100,000 population (before the outbreak period) to 1.12 cases per 100,000 population (during the outbreak period between July 2017 and June 2018). Interestingly, after the outbreak period, only one woman with AHA was reported in the second semester of 2018, and there was no reported case during 2019.

Patients characteristics, male/female (M/F) ratios, and reporting rates are shown in Table 1 and Table 2, respectively. The M/F ratio for ages older than 18 years was 1.4 (7/5) for the period before July 2017 and increased substantially (13/1) for the period after July 2017. All patients reported during the 2017–2018 period were young men (age 32–55 years). Available data on sexual orientation from eight patients of the outbreak period identified four MSM. Six patients diagnosed during the outbreak period had non-Cypriot nationality; however, most of them were residing in Cyprus. Five of the patients reported previous travel to European countries within three months before diagnosis. All patients were negative for HIV and HCV infection by serology, two patients were found to have past HBV infection, and one was diagnosed with syphilis at the same time. One patient with AHA during the first semester of 2017, just before the outbreak, was taking pre-exposure prophylaxis (PrEP) and interestingly developed higher and prolonged transaminasemia (SGOT 4934 U/L, SGPT 6950 U/L, bilirubin 10.46 mg/dL, INR 1.36, γ- GT 337 U/L). One patient, who was simultaneously diagnosed with chronic hepatitis B, developed fulminant hepatic failure and, after referral, had successful liver transplantation. The laboratory findings of this patient the day before transfer were: SGOT 9966 U/L, SGPT 10330 U/L, bilirubin 21.56 mg/dL, INR 5.44, γ- GT 209 U/L, and a model for end-stage liver disease (MELD) score 36. Another patient, with a history of morbid obesity (BMI 47.3 kg/m^2^), developed fulminant hepatitis A and died. This patient arrived late in the hospital in a hepatic coma and died shortly after intubation due to hepatorenal syndrome and unresponsive shock. One female patient developed a second wave of transaminasemia 34 days after discharge, representing a percentage of 3% in this cohort. Fifteen patients of our cohort were hospitalized (including all the 13 patients of the outbreak period) with a mean duration of hospitalization of 8.7 days (±6.6 days).

## 4. Discussion

AHA cases increased in Cyprus between July 2017 and June 2018. Most of these cases were young men. Our data are in agreement with recent reports of hepatitis A outbreaks in Europe [7,8]. M/F ratios and reporting rates per 100,000 population are comparable with the ECDC results for other European countries [12]. Furthermore, a report of an outbreak in the Tel Aviv area, a neighboring region to Cyprus, with the same epidemiological characteristics as the ones reported in the rest of Europe, underline a possible connection [9]. Moreover, a few months before the outbreak, a young male taking PrEP and self-identified as MSM, with a history of recent travel in Europe, was hospitalized with a prolonged course of illness. Such cases have previously been reported elsewhere [10,11].

Our outbreak (between July 2017 and June 2018) occurred later than the rest of the European outbreaks, which took place between June 2016 and May 2017 [12]. This delay could be explained by the geographical characteristics of Cyprus, which is commercially accessible only by air travel, and by its location at the Eastern border of the European Union. However, the first reported cases of the outbreak had traveled to Europe a few months before, which could point toward a connection between the outbreak in Cyprus and ongoing transmission in other European countries. Unfortunately, we could not solidify such a connection due to a lack of data regarding the genotype and the specific subtypes of hepatitis A viruses. The reason for the lack of data is that hepatitis A genotyping is not routinely performed in Cyprus, and samples were not available for further analysis at the time of this analysis. Another limitation is that information on sexual orientation and MSM status may have been undisclosed by some patients, thus potentially underestimating the role of this transmission mode.

As hygiene improves, contact with the virus during the first years of life is not common, and this results in a decline in the natural immunity of the population. Taking into account the low vaccination status of the reported cases in Cyprus, it is reasonable to expect increased susceptibility among young adults. From this and other data, it seems clear that all patients on PrEP should be shown to be Hepatitis A immune or vaccinated. Although hepatitis A vaccination coverage among MSM in Cyprus is not known, according to the recently published results of the EMIS (European men-who-have-sex-with-men Internet survey) survey, hepatitis A vaccination (at least one dose) was reported by approximately 40% of participants [15]. It is also important to note that a national recommendation for vaccination against hepatitis A in MSM was not in effect in Cyprus before the outbreak. However, given the increase in AHA cases, the Cyprus Ministry of Health promptly informed all physicians of the indications for vaccination with a specific mention for MSM, an action that may have contributed to the observed decline of reported AHA cases.

## 5. Conclusions

Despite the good prognosis of AHA, we must acknowledge the slight albeit existent possibility for fulminant hepatitis or acute hepatic failure, especially in patients with predisposing liver disease such as in those with concomitant chronic hepatitis B or C. In our study, one patient died, and another underwent transplantation, making it the first report of such outcomes during an outbreak in Europe recently [12]. Apart from the substantial morbidity, we should not underestimate the economic burden of such outbreaks. In this direction, public health advice should be accessible to populations at risk, following ECDC recommendations. Beyond promoting vaccination in those at risk for HAV transmission and ensuring access to post-exposure prophylaxis with passive and active immunization, it is crucial to inform the MSM community about preventive measures. Communication via social media or leaflets, including instructions about safer sex practices, could prevent future outbreaks. Last, enhanced surveillance and molecular confirmation with phylogenetic analyses of AHA cases are crucial during an outbreak in order to better understand transmission dynamics and thus to inform decisions for timely interventions.

## Figures and Tables

**Table 1:** Demographic data, laboratory findings, and risk factors for patients with acute hepatitis A.

	All Patients (*n* = 33)	Patients before Outbreak (*n* = 20)	Patients during Outbreak (*n* = 13)
Age, mean ± SD	32.7 ±17.4	29.75 ± 20	37.15 ± 11.9
Male sex (%)	26 (78.8)	13 (65)	13 (100)
Cypriot nationality (%)	20 (60.6)	13 (65)	7 (53.8)
Type of Transmission:			
Food borne (%)	4 (12.1)	3 (15)	1 (7.7)
Family (%)	5 (15.2)	5 (25)	0 (0)
MSM ^1^ (%)	4 (12.1)	0 (0)	4 (30.8)
Other (%)	4 (12.1)	4 (20)	0 (0)
Unknown (%)	16 (48.5)	8 (40)	8 (61.5)
Recent travel (%)	19 (57.6)	14 (70)	5 (38.5)
Unvaccinated for hepatitis A (%)	28 (84.9)	15 (75)	13 (100)
Laboratory findings, mean ± SD:			
SGOT (U/L)	3006 ± 2639	2482 ± 1743	3305 ± 3331
SGPT (U/L)	3785 ± 2571	3775 ± 2269	3790 ± 2860
γ- GT (U/L)	296 ± 420	261 ± 88	315 ± 144
Bilirubin (mg/dL)	9.73 ± 6.15	7.3 ± 3.8	10.97 ± 6.48
INR ^2^	1.73 ± 1.3	1.55 ± 0.7	1.83 ±1.6
Admission to hospital (%)	25 (75.8)	12 (60)	13 (100)
Duration of hospitalization (days),			
mean ± SD	8.7 ± 6.6	10.6 ± 9.7	7.3 ± 3.4
Hepatic failure (%)	2 (6.1)	0 (0)	2 (15.4)
Pre-existing liver disease(%)	2 (6.1)	1 (5)	1 (7.7)
Second wave transaminasemia (%)	1 (3)	1 (5)	0 (0)

^1^ MSM: men who have sex with men, ^2^ INR: international normalized ratio.

**Table 2:** Male/female ratios and reporting rates.

Year	2013	2014	2015	2016	2017	2018	2019 ^‡^
Male/female ratio	1	1	0.5	3	6	8	/
Reporting rate ^+^	0.23	* 0.47(1.05)	0.47	0.35	0.7	1.05	/

^+^ Per 100,000 population, * without/(with) the 5 family members of that year, ^‡^ no cases were recorded during 2019.
